# Influence of *MIF* polymorphisms on CpG island hyper-methylation of *CDKN2A* in the patients with ulcerative colitis

**DOI:** 10.1186/s12881-020-01140-9

**Published:** 2020-10-12

**Authors:** Naoko Sakurai, Tomoyuki Shibata, Masakatsu Nakamura, Hikaru Takano, Tasuku Hayashi, Masafumi Ota, Tomoe Nomura-Horita, Ranji Hayashi, Takeo Shimasaki, Toshimi Ostuka, Tomomitsu Tahara, Tomiyasu Arisawa

**Affiliations:** 1 Department of Gastroenterology, Kanazawa Medical University, 1-1, Daigaku, Uchinada-machi, Ishikawa 920-0293 Japan; 2 Department of Gastroenterology, Fujita Health University, 1-98, Dengakugakubo, Kutsukake-cho, Toyoake, 470-1192 Japan; 3 Department of Gastroenterology and Hepatology, Kansai Medical University, 2-5-1 Shin-machi, Hirakata, Osaka, 573-1010 Japan

**Keywords:** Ulcerative colitis, *CDKN2A*, CpG hypermethylation, Macrophage migration inhibitory factor, Genetic polymorphism

## Abstract

**Background:**

*CDKN2A* hypermethylation is among the major events associated with carcinogenesis and is also observed in non-neoplastic colonic mucosa in patients with ulcerative colitis (UC). Macrophage migration inhibitory factor (MIF) plays a crucial role in promoting gastrointestinal inflammation characteristic of UC. The aim of this study is to explore associations between *CDKN2A* methylation status and *MIF* polymorphisms (rs755622 and rs5844572).

**Methods:**

One hundred and fifty-nine patients diagnosed with UC were enrolled in this study. The methylation status of *p14*^*ARF*^ and *p16*^*INK4a*^ was determined by MSP; *MIF* genotypes were identified by PCR-SSCP.

**Results:**

We found no differences with respect to mean age, gender, clinical type (chronic continuous or relapse/remitting), or extent of disease among the patients with methylated and unmethylated *p14*^*ARF*^ or *p16*^*INK4a*^. Carrying the rs755622 C allele indicated a significantly higher risk for *p14*^*ARF*^ methylation (odds ratio (OR), 2.16; 95% confidence interval (CI), 1.08–4.32; *p* = 0.030); similarly, carrying the rs5844572 7-repeat allele indicated a significantly higher risk for *p16*^*INK4a*^ methylation (OR, 2.57; 95% CI, 1.26–5.24; *p* = 0.0094) after an adjusted regression analysis. The carriers of the rs755662 C allele or the rs5844572 7-repeat allele were both at a significantly higher risk for methylation of both *p14*^*ARF*^ and *p16*^*INK4a*^ when compared to the cohort in which neither of the genes were methylated (OR, 2.70; 95% CI, 1.22–6.01; *p* = 0.015 and OR, 2.87; 95% CI, 1.25–6.62; *p* = 0.013, respectively). Additionally, carrying rs755622 C allele was significantly associated with CIHM in chronic continuous of clinical type and total colitis (OR, 25.9; 95% CI, 2.55–262.6; *p* = 0.0059 and OR, 4.38; 95% CI, 1.12–17.2; *p* = 0.034, respectively), and carrying 7-repeat allele of rs5844572 was significantly associated in chronic continuous type (OR, 14.5; 95%CI, 1.46–144.3; *p* = 0.022).

**Conclusions:**

Taken together, our findings suggest that *MIF* genotypes associated with inflammation may also be involved in promoting carcinogenesis via *CDKN2A* hypermethylation in patients diagnosed with UC.

## Background

Ulcerative colitis (UC) is nonspecific inflammation of the large intestine with unknown etiology and its inflammation may involve the colonic mucosa spanning from the rectum to the cecum [1]. UC patients have a chronic or remission/relapsing course and many inflammation- or immune- related factors attribute to the severity of inflammation. Macrophage migration inhibitory factor (MIF) was initially identified as a factor released by T cells that inhibits the random migration of macrophages [2]. Subsequent studies revealed that MIF is a pro-inflammatory factor, which has important roles in various chronic inflammatory diseases and immune disorders, including UC [3, 4]. In particular, Ishiguro et al. reported that MIF contributes to steroid resistance of refractory UC via activator protein (AP)-1 signaling [5]. Two distinct polymorphisms were identified in *MIF*: rs755622 (− 173 G > C) and rs5844572 (− 794 CATT tandem repeat), that were found to be in linkage disequilibrium [6]. Our previous study revealed that these genetic polymorphisms had little influence on the susceptibility to UC [7]; however, a recent meta-analysis based on recessive and co-dominant genetic models identified a significant relationship linking the rs755622 polymorphism and susceptibility to disease [8, 9]. As such, the *MIF* genotype seems to influence the development and progression of UC.

Recent advances with respect to our understanding the pathogenesis of UC together with the development of new therapeutic agents have introduced the possibility of disease control in many cases of UC [10]. However, as the incidence of colitis-associated-cancer (CAC) increases among patients with UC in proportion to the duration of the disease [11, 12], prevention of carcinogenesis and identification of high-risk groups are currently essential clinical issues. Generally, important risk factors for development of CAC are the existence of extensive colonic lesions [11], longer duration of disease [12], positive family history of colorectal cancers [13, 14] and the presence of histologically-active inflammation [15]. However, the risk factors underlying UC-associated carcinogenesis require further and ongoing clarification. CpG island hypermethylation (CIHM) is a critical mechanism that promotes gene inactivation and is commonly observed in association with numerous human cancers [16]. Additionally, CIHM of several specific genes, a phenomenon known as age-related methylation, was also detected in non-neoplastic tissues [17]; this type of methylation has been related to precancerous states [18]. CIHM has been reported within non-neoplastic colonic mucosal tissues of patients diagnosed with UC; likewise, chronic inflammation has been shown to promote age-related methylation [19]. Our previously study also revealed aberrant methylation of the tumor suppressors *p14*^*ARF*^ and *p16*^*INK4a*^, both encoded by Cyclin Dependent Kinase Inhibitor 2A (*CDKN2A*) locus, in the non-neoplastic colonic mucosal tissues of patients with UC [20].

As such, we considered the possibility of identifying patients at high risk for the development of CAC by examining the impact of specific genotypes on CIHM of the genes associated with precancerous states. In the current study, we explored the relationship between polymorphisms of *MIF*, a gene encoding a pro-inflammatory mediator associated with UC, and CIHM of *p14*^*ARF*^ and *p16*^*INK4a*^. Our goal was to determine whether the *MIF* gene polymorphisms have any implications for the assessment of UC patients at high risk for carcinogenesis.

## Methods

### Patients and samples

One hundred and fifty-nine patients with UC were enrolled in this study. All patients were treated at the Endoscopic Center of Fujita Health University Hospital, registered from January 2006 to December 2012. UC was diagnosed according to the standard criteria such as clinical, endoscopic, and histological features [21]. When colonoscopy was performed, the biopsy specimens of inflammatory mucosa were obtained from the rectum of all patients and reserved in − 80 °C. All patients were in endoscopic remission clinically but mild or moderate inflammation without evidence of dysplasia or neoplasia was shown by histopathological examinations showed in all cases. Genomic DNA was isolated using the FlexiGene DNA Kit (QIAGEN GmbH, Hilden, Germany) from peripheral blood obtained at the same time as colonoscopy. The protocol for the present study was approved by the Ethics Committee of Fujita Health University (HM18–094), and written consent was obtained in all cases.

### Classifications

The enrolled patients were classified into two groups, including chronic continuous and relapse/remitting phenotypes, according to their previous clinical course [22]. Patients were also classified by endoscopic features as total or subtotal (distal or left side) colitis according to the location and extent of the inflammatory lesions.

### Detection of DNA methylation of *p14*^*ARF*^ and *p16*^*INK4a*^ by methylation-specific PCR method (MSP)

CIHM of *p14*^*ARF*^ and *p16*^*INK4a*^ was assessed according to the method previously described [23]. We treated genomic DNA, which extracted from rectal biopsy specimens using proteinase K, with sodium bisulfite using the BislFast DNA Modification Kit for methylated DNA Detection (Toyobo, Co., Ltd., Osaka, Japan). The primer sets used at MSP were shown in Table 1. We determined the annealing temperature and times using DNA from peripheral blood of a young individual (as an unmethylated control) and its DNA treated with SssI methylase (methylated control; New England BioLabs Inc., Beverly, MA, USA). Using EX Taq HS (Takara Bio, Shiga, Japan), the PCR was performed with the addition of 0.1 μg of bisulfite-modified DNA in 20 μL of a buffer. The PCR condition were an initial denaturing step of 5 min at 95 °C, followed by 33 cycles of 30 s denaturing at 95 °C, 1 min annealing at 64–68 °C according to primers used, and 1 min extension at 72 °C, and a final 5 min extension step at 72 °C. To detect the band of MSP sample, we performed electrophoresis of PCR products in 3.0% agarose gels stained with ethidium bromide. Then, fluorescence intensity of UV illumination was measured by a digital densitometer. The methylation ratio was calculated as the ratio of intensities of the methylated band to methylated plus unmethylated bands, and a ratio more than 50% was judged as significantly methylated.

**Table 1:** Primer sets used in this study

Primer sets for MSP	
p14-UM_F	5′-gagtttggttttggaggtgg-3’
p14-UM_R	5′-aaccacaacaacaaacacccct-3’
p14-M_F	5′-tgagtttggttttggaggtgg-3’
p14-M_R	5′-aaaaccacaacgacgaacg-3’
p16-UM_F	5′-ttattagagggtggggtggattgt-3’
p16-UM_R	5′-caaccccaaaccacaaccataa-3’
p16-M_F	5′-ttattagagggtggggcggatcgc-3’
p16-M_R	5′-accccgaaccgcgaccgtaa-3’
Primer sets for MIF polymorphism detection	
rs755622_F	5′-tctagccgccaagtggagaaca-3’
rs755622_R	5′-actgtggtcccgccttttgtga-3’
rs5844572_F	5′-tgatccagttgctgccttgtc-3’
rs5844572_R	5′-tccactaatggtaaactcggggac-3’

*MSP* Methylation specific RCR, *UM* Unmethylated, *M* Methylated, *F* Forward, *R* Reverse

### Genotyping of *MIF* polymorphisms

The genotype of *MIF* polymorphisms was determined by the polymerase chain reaction (PCR)-single-strand conformation polymorphism (SSCP) method as described previously [7]. The primer sets used were shown in Table 1. The PCR was performed using EX Taq HS (Takara Bio, Shiga, Japan), adding 0.1 μg of genomic DNA extracted from peripheral blood to 20 μL of a buffer, denaturing at 95 °C for 3 min, followed by 35 cycles of 15 s at 96 °C, 40s at 60 °C for rs755622 or 62 °C for rs5844572, and 30 s at 72 °C, and 5 min final extension at 72 °C. Then, 2 μL of the PCR product was treated in 10 μL of formamide for 5 min at 90 °C and denatured to a single strand. SSCP was performed in Gene Phor DNA separation system using the Gene Gel Excel 12.5/24 kit (GE Health Care Bio-Sciences AB, Stockholm, Sweden) at a constant temperature of 6 °C, and the denatured bands were detected using the DNA silver staining kit (GE Health Care Bio-Sciences AB).

### Statistical analysis

The Hardy–Weinberg equilibrium (HWE) was assessed by χ^2^ statistics. Mean age was expressed as mean ± SD and analyzed by Student’s t-test. The ratio of sex and CIHM frequencies was compared by Fisher’s exact test. Allele counts and genotype distribution were also compared between two groups by Fisher’s exact test. The odds ratio (OR) and 95% confidence intervals (CI) for the strength of genotype involvement in CIHM were calculated using a logistic regression analysis adjusted for age, sex, clinical type and disease extension. A probability value of less than 0.05 was considered statistically significant in all analyses. Stata software (version 13; StataCorp LP, College Station, TX, USA) was used for statistical processing.

## Results

### Demographic characteristics, allelic frequencies, and *CDKN2A* methylation status

The characteristics and allele frequencies observed among the UC patients enrolled in this study are shown in Table 2. The allele distribution of *MIF* (rs755622) met the criteria for HWE (*p* = 1.00). We found no differences with respect to mean age, gender, clinical type, or extent of disease among those with methylated and unmethylated *p14*^*ARF*^ or *p16*^*INK4a*^. The minor allele frequency of rs755622 was somewhat higher in the group with *p14*^*ARF*^ methylation; of note, the frequency of the C allele carrier was significantly higher (*p* = 0.01). By contrast, no significant differences in the minor allele frequencies associated with rs755622 were observed when comparing the *p16*^*INK4a*^ methylated and unmethylated groups. Similarly, the 7-repeat allele frequency of rs5844572 was significantly higher in the *p16*^*INK4a*^ methylated group compared to unmethylated group (*p* = 0.036), but no significant differences were observed when comparing the *p14*^*ARF*^ methylated with unmethylated groups.

**Table 2:** Demographic characteristics, allelic frequencies, and *CDKN2A* methylation status

	Overall UC	p14-UM	p14-M	p^a^	p16-UM	p16-M	p^b^
Number of sample	159	105	54		89	70	
Mean age ± SD	41.3 ± 13.6	40.4 ± 13.4	43.0 ± 14.0	NS	42.1 ± 15.2	40.2 ± 11.3	NS
Male: female	91: 68	66: 39	25: 29	NS	50: 39	41: 29	NS
Clinical type				NS			NS
Chronic continuous	56	38	18		31	25	
Relapse/remitting	103	67	36		58	45	
Disease extension				NS			NS
Total colitis	74	49	25		35	39	
Distal or left side colitis (rs755622 G > C)	85	56	29		54	31	
GG	93	68	25	0.010	57	36	NS
GC	57	32	25		28	29	
CC	9	5	4	NS	4	5	NS
C allele freqency (rs5844572 CATT repeat)	23.6%	20.0%	30.6%	0.050	20.2%	27.9%	NS
5/5	16	13	3		9	7	
5/6	57	40	17		31	26	
5/7	28	16	12		12	16	
6/6	28	19	9		23	5	
6/7	23	14	9		12	11	
7/7	7	3	4		2	5	
5 repeat freqency	36.8%	39.0%	32.4%		34.3%	40.0%	
6 repeat freqency	42.8%	43.8%	40.7%		50.0%	42.1%	
7 repeat freqency	20.4%	17.1%	26.9%	NS	15.7%	26.4%	0.036

p14-UM, *p14*^*ARF*^ unmethylated; p14-M, *p14*^*ARF*^ methylated; p16-UM, *p16*^*INK4a*^ unmethylated;

p16-M, *p16*^*INK4a*^ methylated; ^a^p14-UM vs. p14-M; ^b^p16-UM vs. p16-M; *NS* Not significant

### Association between *MIF* polymorphisms and methylation status of *p14*^*ARF*^ or *p16*^*INK4a*^

By a logistic regression analysis after adjusting for confounding factors including age, gender, clinical type, and extent of disease, carrying C allele of rs755622 was significantly associated with CIHM of *p14*^*ARF*^ (Table 3; OR, 2.16; 95% CI, 1.08–4.32; *p* = 0.030). By contrast, no significant relationship was found between *p16*^*INK4a*^ methylation and the allele frequencies associated with rs755622. The rs755622 CC homozygous was not associated with CIHM of both *p14*^*ARF*^ and *p16*^*INK4a*^by a recessive genetic model.

**Table 3:** Association between *MIF* rs755622 and *CDKN2A* methylation

	Genotype			GG vs. GC + CC	GG + GC vs. CC
	GG	GC	CC	adjusted OR* (95%CI); *p* value	adjusted OR* (95% CI); *p* value
p14-UM (105)	68	32	5	reference	reference
p14-M (54)	25	25	4	2.16 (1.08–4.32); *p* = 0.030	2.30 (0.52–10.3); *p* = 0.27
p16-UM (89)	57	28	4	reference	reference
p16-M (70)	36	29	5	1.90 (0.974–3.69); *p* = 0.060	2.25 (0.52–9.69); *p* = 0.28

*by logistic regression analysis after adjustment for age, gender, clinical type and disease extension

*UM* Unmethylated, *M* Methylated

We previously revealed that CATT 7-repeat allele of rs5844572 promotes inflammation. Thus, we assessed the influence of the 7-repeat allele. Our findings indicate that carrying the rs5844572 7-repeat allele was a significant risk factor for *p16*^*INK4a*^ methylation by an adjusted logistic regression analysis (Table 4; OR, 2.57; 95% CI, 1.26–5.24; *p* = 0.0094). By contrast, there were no significant relationships between *p14*^*ARF*^ methylation and rs5844572 allele frequencies. The homozygous of rs5844572 7-repeat allele was not associated with CIHM of both *p14*^*ARF*^ and *p16*^*INK4a*^.

**Table 4:** Association between *MIF* rs5844572 and *CDKN2A* methylation

	Genotype (repeat number)						X/X vs. X/7 + 7/7	X/X + X/7 vs. 7/7
	5/5	5/6	5/7	6/6	6/7	7/7	adjusted OR* (95% CI); *p* value	adjusted OR* (95% CI); *p* value
p14-UM (105)	13	40	16	19	14	3	reference	reference
p14-M (54)	3	17	12	9	9	4	1.77 (0.864–3.63); *p* = 0.12	4.51 (0.79–25.7); *p* = 0.090
p16-UM (89)	9	31	12	23	12	2	reference	reference
p16-M (70)	7	26	16	5	11	5	2.57 (1.26–5.24); *p* = 0.0094	5.51 (0.90–33.9); *p* = 0.066

*by logistic regression analysis after adjustment for age, gender, clinical type and disease extension

*UM* Unmethylated, *M* Methylated, X: 5 or 6 repeat allele; 7: 7 repeat allele

### Demographic characteristics and allele frequencies of subjects demonstrating no methylation or methylation of both *p14*^*ARF*^ and *p16*^*INK4a*^

Comparisons among groups demonstrating methylation of both *p14*^*ARF*^ and *p16*^*INK4a*^ with those in which both were unmethylated are shown in Table 5. The allele distribution of *MIF* (rs755622) in both methylated and neither methylated groups met the criteria for HWE (*p* = 0.73 and *p* = 0.72, respectively). There were no significant differences with respect to clinicopathological backgrounds between these two groups.

**Table 5:** Demographic characteristics and allele frequencies of subjects demonstrating no methylation or methylation of both *p14*^*ARF*^ and *p16*^*INK4a*^

	Neither methylated	Both methylated	*p* value
Number of sample	77	42	
Mean age ± SD	41.6 ± 14.9	42.5 ± 12.9	NS
Male: female	46: 31	21: 21	NS
Clinical type			NS
Chronic continuous	25	12	
Relapse/remitting	52	30	
Extension			NS
Total colitis	31	21	
Distal or left side colitis (rs755622 G > C)	46	21	
GG	49	17	0.020
GC	24	21	
CC	4	4	NS
C allele freqency (rs5844572 CATT repeat)	20.8%	34.5%	0.029
5/5	7	1	
5/6	27	13	
5/7	12	12	
6/6	19	5	
6/7	10	7	
7/7	2	4	
5 repeat freqency	34.4%	32.1%	
6 repeat freqency	48.7%	35.7%	
7 repeat freqency	16.9%	32.1%	0.0090

*p* value: unmethylated vs. both methylated

The minor allele frequencies associated with rs755622 were significantly higher in the group in which both *p14*^*ARF*^ and *p16*^*INK4a*^ were methylated compared to the fully unmethylated group (*p* = 0.029); the C allele carrier was detected at significantly higher frequency (*p* = 0.020). Similarly, the frequency of the rs5844572 7-repeat allele was significantly higher in the group in which both *p14*^*ARF*^ and *p16*^*INK4a*^ were methylated compared to the fully unmethylated group (*p* = 0.0090).

### Association between *MIF* polymorphisms and *CDKN2A* methylation

The results of an analysis in which confounding factors were adjusted revealed that carrying the rs755622 C allele and the rs5844572 7-repeat allele was significantly associated with an increased methylation of both *p14*^*ARF*^ and *p16*^*INK4a*^ (OR, 2.70; 95% CI, 1.22–6.01; *p* = 0.015 and OR, 2.87; 95% CI, 1.25–6.62; *p* = 0.013, respectively; Table 6). In addition, homozygous of rs5844572 7-repeat allele was significantly associated with CIHM of both genes (OR, 12.0; 95%CI, 1.55–92.2; *p* = 0.017).

**Table 6:** Association between *MIF* polymorphisms and *CDKN2A* methylation

rs755622	GG vs. GC + CC	GG + GC vs. CC
	adjusted OR* (95% CI); *p* value	adjusted OR* (95% CI); *p* value
Neither methylated (77)	reference	reference
Both methylated (42)	2.70 (1.22–6.01); *p* = 0.015	3.92 (0.76–20.3); *p* = 0.10
rs5844572	X/X vs. X/7 + 7/7	X/X + X/7 vs. 7/7
	adjusted OR* (95% C.I.); *p* value	adjusted OR* (95% C.I.); *p* value
Neither methylated (77)	reference	reference
Both methylated (42)	2.87 (1.25–6.62); *p* = 0.013	12.0 (1.55–92.2); *p* = 0.017

*by logistic regression analysis after adjustment for age, gender, clinical type and disease extension

X: 5 or 6 repeat allele; 7: 7 repeat allele

### Association between *MIF* polymorphisms and *CDKN2A* methylation in phenotype of UC

Next, we investigated in what kind of UC phenotype the significant association of *MIF* polymorphisms with CIHM of *CDKN2A* was seen (Table 7). Carrying rs755622 C allele was significantly associated with CIHM in chronic continuous of clinical type and total colitis (OR, 25.9; 95% CI, 2.55–262.6; *p* = 0.0059 and OR, 4.38; 95% CI, 1.12–17.2; *p* = 0.034, respectively). Meanwhile, carrying 7-repeat allele of rs5844572 was significantly associated in chronic continuous type (OR, 14.5; 95%CI, 1.46–144.3; *p* = 0.022).

**Table 7:** Association between *MIF* polymorphisms and *CDKN2A* methylation in UC phenotype

rs755622	genotype			adjusted OR (95% C.I.); *p* value
Chronic continuous	GG	GC	CC	GG vs. GC + CC
Neither methylated (25)	17	6	2	reference
Both methylated (12)	2	6	4	25.9 (2.55–262.6); *p* = 0.0059^a^
Relapse/remitting	GG	GC	CC	GG vs. GC + CC
Neither methylated (52)	32	18	2	reference
Both methylated (30)	15	15	0	1.93 (0.732–5.07); *p* = 0.18^a^
Total colitis	GG	GC	CC	GG vs. GC + CC
Neither methylated (31)	21	10	0	reference
Both methylated (21)	9	12	0	4.38 (1.12–17.2); *p* = 0.034^b^
Distal or left side colitis	GG	GC	CC	GG vs. GC + CC
Neither methylated (46)	28	14	4	reference
Both methylated (21)	8	9	4	2.75 (0.899–8.43); *p* = 0.076^b^
rs5844572	genotype			adjusted OR (95% C.I.); *p* value
Chronic continuous	X/X	X/7	7/7	X/X vs. X/7 + 7/7
Neither methylated (25)	15	8	2	reference
Both methylated (12)	3	5	4	14.5 (1.46–144.3); *p* = 0.022^a^
Relapse/remitting	X/X	X/7	7/7	X/X vs. X/7 + 7/7
Neither methylated (52)	38	14	0	reference
Both methylated (30)	16	14	0	2.64 (0.970–7.17); *p* = 0.058^a^
Total colitis	X/X	X/7	7/7	X/X vs. X/7 + 7/7
Neither methylated (31)	21	10	0	reference
Both methylated (21)	10	11	0	3.47 (0.895–13.4); *p* = 0.072^b^
Distal or left side colitis	X/X	X/7	7/7	X/X vs. X/7 + 7/7
Neither methylated (46)	32	12	2	reference
Both methylated (21)	9	8	4	2.89 (0.931–8.98); *p* = 0.066^b^

^a^adjusted for age, gender and disease extension

^b^adjusted for age, gender and clinical type

X: 5 or 6 repeat allele; 7: 7 repeat allele

## Discussion

In the present study, we investigated the impact of *MIF* gene polymorphisms on aberrant methylation in the promoter regions of *p14*^*ARF*^ and *p16*^*INK4a*^, each generated by alternative splicing at the *CDKN2A* locus, in a cohort of 159 patients diagnosed with UC. Our results revealed that *MIF* rs755622 C and rs5844572 7-repeat alleles were associated with *p14*^*ARF*^ and *p16*^*INK4a*^ methylation, respectively. Furthermore, the rs755622 C and rs5844572 7-repeat alleles were both associated with enhanced *CDKN2A* methylation among patients in which both *p14*^*ARF*^ and *p16*^*INK4a*^ were methylated compared to those in which neither of the sites were methylated using a dominant genetic model. We suspect that no significant association between homozygous of both genotypes and CIHM of *CDKN2A* based on a recessive genetic model may be due to a small number of subjects in this study.

MIF is a proinflammatory cytokine that promotes recruitment of neutrophils and macrophages to inflammatory foci in the setting of inflammatory disease [24]. Several studies have focused on MIF as a key molecule promoting pathogenesis of a diverse array of diseases, including rheumatoid arthritis [25] and septic shock [26]. MIF is also a critical mediator of UC [4, 5, 27]. Renner et al. reported that polymorphisms in the human *MIF* gene were associated with susceptibility to and severity of several inflammatory diseases, including UC [6]. Likewise, Donn et al. revealed by promoter sequence analysis that change of G to C at − 173 (rs755622) has a direct impact on MIF expression as it creates a potential binding site for the transcription factor, AP-4; transcriptional activity of the *MIF* gene increases in accordance with the number of sequence repeats associated with the rs5844572 polymorphism [28]. Similarly, Amoli et al. reported that a *MIF* promoter with rs5844572 5-repeat was less transcriptionally active than those with 6- and 7-repeats [29]. In GTEx portal site (https://gtexportal.org/), an increased number of rs755622 minor allele correlates to the increased expression of MIF, although the data of rs5844572 is not shown. Since the rs755622 C and rs5844572 7-repeat alleles are in strong linkage disequilibrium [6], the combination of these two alleles may constitute an inflammatory haplotype. This is consistent with the reported associations linking the rs755622 C- and rs5844572 7-repeat haplotype with susceptibility to juvenile idiopathic arthritis [28] as well as to findings in our previous studies focused on gastric inflammation and carcinogenesis [30, 31]. However, in our previous study [7], genetic polymorphisms in *MIF* were not strongly associated with susceptibility to UC; likewise, Nohara et al. reported no differences with respect to the distribution of the rs755622 genotype when comparing findings from healthy subjects to those diagnosed with UC patients from the general Japanese population [32]. The results of these studies suggest that the proinflammatory haplotype of *MIF* may not be significantly involved in susceptibility to UC in the Japanese population.

The p14^ARF^ and p16^INK4a^ proteins are encoded by *CDKN2A* by alternative splicing; these proteins act on the p53 and pRb pathways, respectively, to promote negative regulation of the cell cycle [33, 34]. As such, methylation-mediated silencing of gene expression may have important implications with respect to carcinogenesis. Poi et al. have shown that methylation at each promoter site has resulted in gene deletion or silencing in association with several cancers [35]. Conversely, gene methylation has been associated with chronic inflammation [36], and methylation of both *p14*^*ARF*^ and *p16*^*INK4a*^ is already enhanced in non-neoplastic colonic mucosa of patients with UC [37, 38]. Of these two loci, methylation at *p16*^*INK4a*^ seems to be of greater importance with respect to the development of CAC [39]. However, methylation at *p14*^*ARF*^ may also have important implications; methylation of both *p14*^*ARF*^ and *p16*^*INK4a*^ was reported among the more invasive forms of sporadic colorectal cancer [40]. Our present observations revealed a significant relationship between the proinflammatory allele of *MIF* and methylation of both *p14*^*ARF*^ and *p16*^*INK4a*^ in the colonic mucosa of patients diagnosed with UC. These findings stand in contrast to those reported in our previous study [7], in which we found that these alleles were not significantly associated with susceptibility to UC. Taken together, we infer from these results that carrying the proinflammatory allele of *MIF* may be involved in the intensity of inflammation observed after the onset of UC among those in the Japanese population; this allele may not be involved in the development of UC, but is involved in promoting *CDKN2A* methylation. Although it is unclear whether methylation at these sites is directly involved in the development of CAC in patients with UC, it remains possible that individuals carrying an inflammatory allele in *MIF* may be at higher risk for this complication. Furthermore, in our results, the significant association of *MIF* polymorphisms with CIHM of *CDKN2A* were found in chronic continuous of clinical type and total colitis phenotype. Rogler has been reported continuous severe inflammation is involved in the development of CAC in UC [41]. Meanwhile, it is well known that the extent of inflammatory colonic mucosa is related to the increased risk for the development of CAC in UC [12]. These facts suggest that the patients with chronic continuous phenotype and total colitis of UC have a higher risk for development of CAC than with relapse/remitting phenotype and left sided/ distal colitis of UC, respectively. *MIF* polymorphisms may contribute to further increasing the high risk for the development of CAC via CIHM of *CDKN2A*.

In our present study, we focused on the CIHM of *CDKN2A*. However, there is possibility that CpG islands of many other genes are methylated in inflammatory mucosa of UC. Recently, Tahara, one of the co-authors in this study, revealed a high rate of hypermethylation in the severe phenotype of UC, particularly at the CpG islands, by genome-wide methylation analysis, and that these methylated genes were related to those involved in biosynthetic processes, the regulation of metabolic processes, and nitrogen compound metabolic processes [42]. In addition, we have already reported that function gain genotypes of various immune- or inflammation-related molecules were associated with an increased CpG methylation of *CDH1*, encoding e-cadherin, and *CDKN2A* [20, 43]. Further studies for an association of various genotypes with CpG islands methylation of the responsible genes for development of CAC will be needed.

There are various limitations to this study. First, the study a retrospective and utilized previously-stored tissue samples collected at a single institution in Japan. A multi-centered prospective study based on these findings should be conducted in the near future. Second, as this study was conducted using a small number of samples, we were unable to examine other gene polymorphisms that might influence the methylation status of the *MIF* gene. As above, a multi-centered study may provide more samples for evaluation. Third, patients enrolled in this study have taken various medications, not the same medications. In addition, since the onset age of our patients was partially unclear, the analysis could not be performed using disease duration as a co-variate. Finally, a full evaluation of the risk of developing CAC from UC would include patients with CAC as well as those diagnosed with a precancerous condition. Again, due to the very limited number of samples from patients who developed CAC at our institution, we were unable to study this phenomenon directly. As such, we included samples from patients with precancerous conditions as a next best practice.

## Conclusions

In conclusion, our findings indicate that the rs755622 C–rs5844572 7-repeat *MIF* haplotype, which includes two distinct alleles that are in strong linkage disequilibrium, is significantly associated with increased methylation of both *p14*^*ARF*^ and *p16*^*INK4a*^. These observations suggest that UC patients with this inflammatory genotype of *MIF* may be at a higher risk for developing CAC.

## Data Availability

All data generated during this study are included in this published article. The raw data analyzed during the current study are not publicly available due to risk of compromising individual privacy. The application and the written consent forms state that the data will only be available to the researchers within the project. For inquires on the data, researchers should first reach out to the owner of the database, Fujita Health University. Please reach out to the corresponding author with requests and for assistance with data requests.

## Abbreviations

UC: Ulcerative colitis
MIF: Macrophage migration inhibitory factor
AP: Activator protein
CAC: Colitis-associated-cancer
CIHM: CpG island hyper-methylation
CDKN2A: Cyclin Dependent Kinase Inhibitor 2A
PCR: Polymerase chain reaction
MSP: Methylation-specific RCR
PCR-SSCP: PCR- single-strand conformation polymorphism
HWE: Hardy–Weinberg equilibrium
OR: Odds ratio
CI: Confidence interval
pRB: Retinoblastoma protein.
